# Isoquercitrin Attenuates Steatohepatitis by Inhibition of the Activated NLRP3 Inflammasome through HSP90

**DOI:** 10.3390/ijms24108795

**Published:** 2023-05-15

**Authors:** Ji Ma, Maoru Li, Tingting Yang, Yang Deng, Yadong Ding, Tiantian Guo, Jing Shang

**Affiliations:** 1 School of Traditional Chinese Pharmacy, China Pharmaceutical University, Nanjing 211198, China; matthewmj@yeah.net (J.M.); guotiantian2000@outlook.com (T.G.); 2 Jiangsu Key Laboratory of TCM Evaluation and Translational Research, China Pharmaceutical University, Nanjing 211198, China

**Keywords:** isoquercitrin, metabolic-associated fatty liver disease, NLRP3 inflammasome, heat shock protein 90

## Abstract

Non-alcoholic fatty liver disease (NAFLD) is a chronic liver disease with a global prevalence of 25%. However, the medicines approved by the FDA or EMA are still not commercially available for the treatment of NAFLD. The NOD-like receptor thermal protein domain-associated protein 3 (NLRP3) inflammasome plays a crucial role in inflammatory responses, and the mechanisms related to steatohepatitis have been sufficiently clarified. NLRP3 has been widely evaluated as a potential target for multiple active agents in treating NAFLD. As a quercetin glycoside, isoquercitrin (IQ) has a broad inhibitory effect on oxidative stress, cancers, cardiovascular diseases, diabetes, and allergic reactions in vitro and in vivo. This study aimed to investigate the undercover mechanism of IQ in the treatment of NAFLD, particularly in anti-steatohepatitis, by suppressing the NLRP3 inflammasome. In this study, a methionine-choline-deficient induced steatohepatitis mice model was used to explore the effect of IQ on NAFLD treatment. Further mechanism exploration based on transcriptomics and molecular biology revealed that IQ inhibited the activated NLRP3 inflammasome by down-regulating the expression of heat shock protein 90 (HSP90) and suppressor of G-two allele of Skp1 (SGT1). In conclusion, IQ could alleviate NAFLD by inhibiting the activated NLRP3 inflammasome by suppressing the expression of HSP90.

## 1. Introduction

Metabolic-associated fatty liver disease (MAFLD) is a chronic liver disease that has been renamed from non-alcoholic fatty liver disease (NAFLD) in recent years. The global prevalence of NAFLD is approximately 25%. It has brought an onerous burden on the healthcare system worldwide [1,2]. NAFLD includes a pathological spectrum of simple steatosis, steatohepatitis, and hepatocellular carcinoma (HCC). The progression of simple steatosis can be reversed through a modified diet or exercise. However, without medication, steatohepatitis is irreversible and can develop into HCC. Moreover, steatohepatitis is considered one of the leading etiological factors of HCC and a suitable indication for liver transplantation [3,4]. Currently, there is still no medicine approved by the FDA or EMA on the market to treat steatohepatitis.

Steatohepatitis derives from simple steatosis and occurs when an overload lipid accumulation exceeds the capacity of hepatocytes, resulting in lipotoxicity, lipid peroxidation, inflammation, and oxidative stress that can lead to a lethal hepatocyte injury. Recently, the focus of most steatohepatitis studies has been placed on immune responses induced by factors produced by injured hepatocytes, such as reactive oxygen species (ROS), inflammatory cytokines, and cholesterol crystals [5]. These endogenous factors can be recognized by danger-associated molecular patterns (DAMPs). In addition, they can induce further inflammatory activation through a NOD-like receptor (NLR) [6,7]. The NOD-like receptor thermal protein domain-associated protein 3 (NLRP3) inflammasome is one of the key NLR inflammasomes in Kupffer cells (KCs) and hepatocytes. As a large intracellular complex, NLRP3 consists of a sensor and an adaptor apoptosis-associated speck-like protein (ASC) [8]. The activated NLRP3 inflammasome in KCs can promote caspase-1 maturation, which further cleaves pro-IL-1β into mature forms and leads to its secretion [9] (known as the M1 polarization). Secreted IL-1β accelerates immune responses by toll-like receptors (TLRs) in hepatocytes and KCs through pathogen-associated molecular patterns (PAMPs). This may even induce fibrosis from steatohepatitis until the injured factors can be controlled or M2 KCs can be polarized [10,11]. Further, the NLRP3 inflammasome plays a crucial role in inflammatory responses, and the mechanisms related to steatohepatitis have been sufficiently clarified. NLRP3 has been widely evaluated as a potential target for multiple active agents in treating NAFLD/steatohepatitis [12,13].

Quercetin (3,3,4′,5,7-pentahydroxyflavone) is an abundant polyphenolic flavonoid with anti-inflammatory, anti-apoptotic, immune-protective, and anti-cancer effects [14]. Meanwhile, it has been suggested that quercetin provides hepatoprotection at different stages of NAFLD in vivo and in vitro [15]. As a natural phytochemical substance with low toxicity and few side effects, quercetin is considered a favorable dietary supplement for NAFLD treatment [16]. Quercetin is also known to exert the effect through various mechanisms of action in the treatment of multiple liver diseases. These mechanisms include inflammation-related NF-kB, TLR and NLRP3, PI3K and its associated Nrf2 and mTOR, and PPAR-associated lipid metabolic pathways. Among these mechanisms, PI3K is involved in insulin resistance, autophagy, and apoptosis. In addition, it also actively participates in lipid metabolism regulation at an early stage of NAFLD. NF-kB, TLR, and NLRP3 are involved in inflammation-related regulation of the liver at an advanced stage of NAFLD. However, the uptake, distribution, metabolism, and excretion of quercetin has been explored from the perspective of bioavailability over the past decades. The results have shown the poor uptake and rapid metabolism of quercetin, which severely limits its further application [17]. A better bioavailability was revealed in quercetin glycosides due to their higher water solubility compared with quercetin. As one of the quercetin glycosides, isoquercitrin (quercetin-3-O-b-D-glucopyranoside, IQ) has received much attention [18]. The results have demonstrated that the bioavailability of IQ was twice that of quercetin in rats, beagles, and humans [19,20]. IQ has a broad inhibitory effect on oxidative stress, cancers, cardiovascular diseases, diabetes, and allergic reactions in vitro and in vivo [21]. In addition, much attention has been paid to IQ due to its high availability. Notably, a product called “enzymatically modified IQ (EMIQ)” has been approved by the FDA for food production [22]. Based on that, our group performed a study on the anti-NAFLD effects of IQ a few years ago [23]. It was found that IQ reduced hepatic lipid accumulation by activating the AMPK pathway and inhibiting TGF-β signaling. Additionally, IQ-rich foods have also been verified to play a role in insulin resistance and lipid metabolism through the PI3K pathway. These results suggest that IQ plays an active role in lipid regulation at an early stage of NAFLD. However, it remains unclear whether IQ also plays a role at a more advanced stage of steatohepatitis. Based on the evidence from the quercetin, we hypothesized that IQ may also exert an anti-inflammatory effect at a more advanced stage of NAFLD. It is necessary to further clarify the mechanism related to anti-inflammatory effects.

According to the positive effect of IQ at an early stage of NAFLD and the pathogenesis of steatohepatitis, this study aimed to investigate the undercover mechanism of IQ in anti-steatohepatitis through immune responses related to NLRP3 inflammasome activation in vivo and in vitro.

## 2. Results

### 2.1. Effects of IQ on the MCD-Induced Steatohepatitis Mice Model

**Figure 1:**
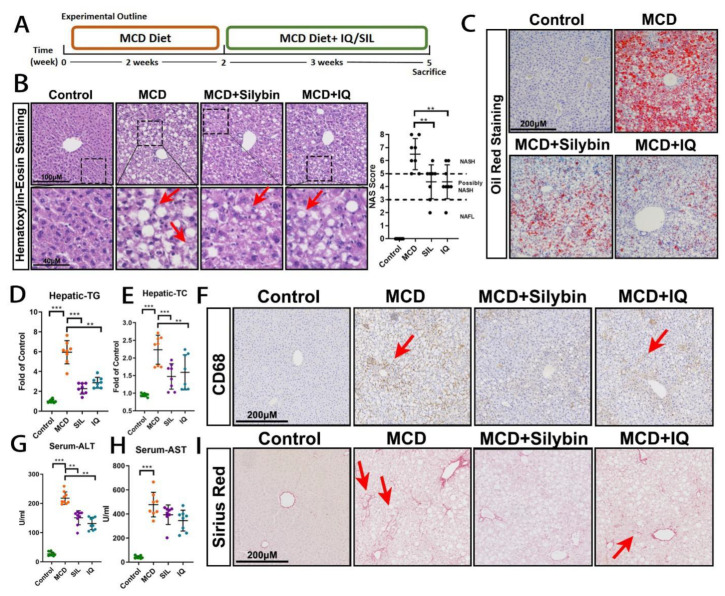
**Effects of IQ on the MCD-induced steatohepatitis mice model.** (**A**) Experimental outline of the MCD-induced steatohepatitis mice model. (**B**) HE staining and the NAS score of liver tissues (steatosis is indicated by red arrows). (**C**) Oil red staining of liver tissues. (**D**) Hepatic TG level of mice (n = 8). (**E**) Hepatic TC level of mice (n = 8). (**F**) Macrophage CD68 staining of liver tissues (CD68+ is indicated by red arrows). (**G**) Serum ALT level of mice (n = 8). (**H**) Serum AST level of mice (n = 8). (**I**) Collagen Sirius Red staining of liver tissues (collagen + is indicated by red arrows). The bar indicates mean ± SD. ** *p* < 0.01, or *** *p* < 0.001 represents the significance of differences, and *p* < 0.05 is considered statistically significant. The significance is calculated using ANOVA, followed by Turkey’s test (n = 8, n indicates the number of animals).

To investigate the anti-steatohepatitis effect of IQ, a classic steatohepatitis model, namely the methionine-choline-deficient (MCD) diet-induced mice model, was conducted (Figure 1A). Specifically, silibinin (SIL) was administrated to mice as a positive control based on its application in the treatment of chronic hepatitis. In contrast, SIL (54.6 mg/kg) and IQ (25 mg/kg) were administered together with an MCD diet for 3 weeks after an MCD diet solely induced mice for 2 weeks. The weight and serum triglyceride (TG) and total cholesterol (TC) were tested. The results indicated no significant change in both the SIL and IQ groups compared with the MCD group (Figure S1A–C). In this model, the serum TG and TC were reduced after it was induced by the MCD diet compared with the control group. This was a characteristic of this model, and it would be difficult to examine the effect of the medicines on relevant serum indexes. As per the HE staining results of liver tissues (Figure 1B), macrovesicular steatosis can be widely found in the MCD group. In addition, possible blooming degeneration was revealed in section samples, which was a typical characteristic of steatohepatitis. Both SIL and IQ reversed the macrovesicular steatosis and degeneration of hepatocytes compared with the MCD group. Moreover, from the non-alcoholic fatty liver disease activity score (NAS) on the HE staining, all animals in the MCD group had a score of >5. This indicated that a typical NASH animal model was established in this study. However, the score of SIL and IQ was significantly lower than that of MCD, which further confirmed that IQ could have an anti-steatohepatitis effect. Oil red staining for steatosis of the liver (Figure 1C) and biochemical measurement of hepatic TG and TC (Figure 1D,E) further proved the positive effect of both SIL and IQ on reducing lipid accumulation in the MCD-induced steatohepatitis mice model. These results verified the positive effects of IQ on anti-NAFLD, which was similar to the finding in a previous study based on an HFD-induced rat model [23]. To further investigate the anti-inflammatory effects of IQ, the CD68 antibody was employed to immunohistochemically stain the macrophages or KCs in the liver. Both the staining results (Figure 1F) and quantitative results using Western blotting (Figure S1D) showed that the number of KCs in both the SIL and IQ groups was lower than that in the MCD group. This indicated that there was milder inflammation in both the SIL and IQ groups than that in the MCD group. A lower level of serum AST and serum ALT (Figure 1G,H) in both the SIL and IQ groups further proved the protective effects of SIL and IQ on the liver. Moreover, collagen Sirius Red staining was performed to investigate the degeneration of steatohepatitis to fibrosis. The results (Figure 1I) revealed that limited collagen was stained in the MCD group, which indicated fibrosis may progress even though the pseudo lobules were not typical. However, there was less collagen in both the SIL and IQ groups. This corroborated that SIL and IQ reduced the progression of fibrosis.

### 2.2. Effects of IQ on Anti-Oxidative Stress and Anti-Inflammation

**Figure 2:**
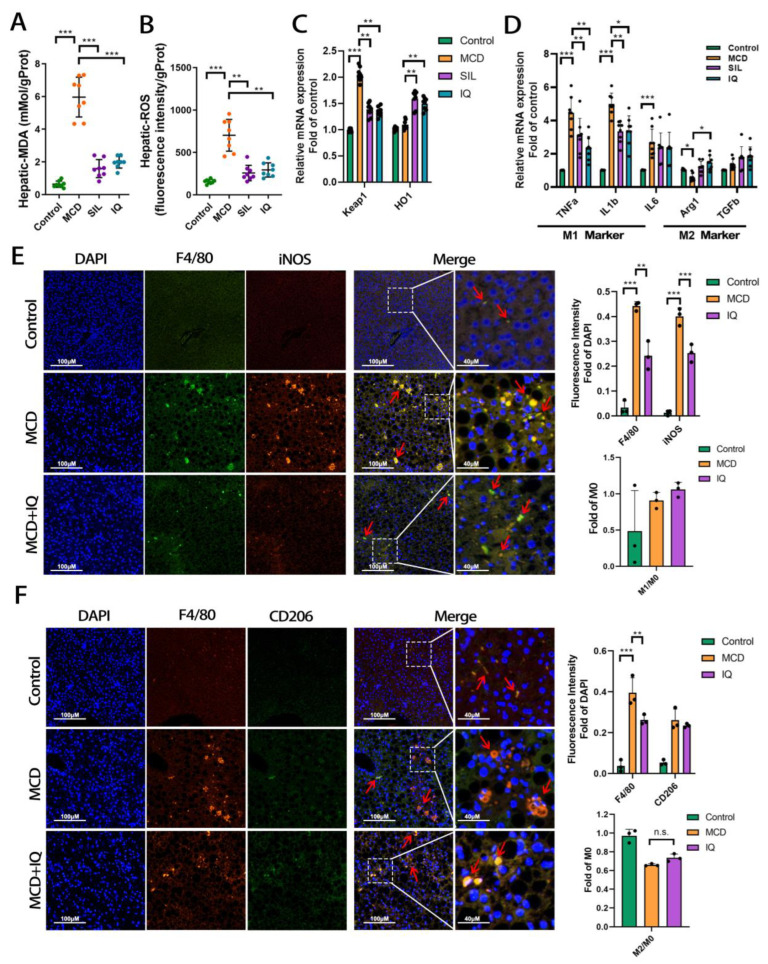
**Effects of IQ on anti-oxidative stress and anti-inflammation.** (**A**) Hepatic MDA of mice (n = 8, n indicates the number of animals). (**B**) Hepatic ROS of mice (n = 8, n indicates the number of animals). (**C**) Relative mRNA expression of oxidative stress (n = 8, n indicates the number of samples). (**D**) Relative mRNA expression of KC polarization (n = 8, n indicates the number of samples). (**E**) Immunofluorescence of KCs after M1 polarization (M1 KCs are indicated by red arrows). (**F**) Immunofluorescence of the polarization of KCs after M2 polarization (M2 KCs are indicated by red arrows). The bar indicates mean ± SD. n.s. *p >* 0.05, * *p* < 0.05, ** *p* < 0.01, *** *p* < 0.001 represents the significance of differences, and *p* < 0.05 is considered statistically significant. The significance is calculated using ANOVA, followed by Turkey’s test or the Kruskal–Wallis test for the nonparametric test.

Given the liver-protecting effect of IQ by reducing KCs and lowering related injury indexes, it is necessary to explore the underlying mechanism. Oxidative stress is the consequence of an overload of lipids in hepatocytes or it can be induced by inflammation. MDA is the end product of lipotoxicity. The level of ROS and MDA increased significantly in the MCD group compared with the control group (Figure 2A,B). Both SIL and IQ reduced the level of MDA and ROS dramatically. The lowering effect on MDA and ROS revealed an anti-oxidative stress effect of IQ. KEAP1 and HO-1 are the two key mRNAs in regulating the anti-oxidative stress effect in the organism. The results showed that the relative mRNA expression of keap 1 was down-regulated, and HO-1 increased in the IQ group. This further confirmed the anti-oxidative stress effect of IQ (Figure 2C). To further investigate the effect of IQ on anti-inflammation, we tested the KCs in the liver to identify the inflammatory reaction. We also measured the mRNA expression of KCs after M1 and M2 polarization in the liver tissue of mice (Figure 2D). It was found that TNFa, IL1b, and IL6 M1 markers were significantly reduced in the SIL and IQ groups compared with the MCD group. However, the increase in Arg1 and TGFb M2 markers was not significant at the mRNA level. Furthermore, the M1 and M2 polarization of KCs was measured by immunofluorescence. The F4/80 antibody was employed to mark the total KCs (M0 type), the iNOS antibody to mark the M1 type, and CD206 to mark the M2 type. The results showed that iNOS expression was significantly down-regulated in the IQ group compared with the MCD group, which was similar to the results of CD68 in Figure 1F. Notably, from the ratio of M1/M0, it can be summarized that almost all M0 KCs were polarized to M1 KCs (Figure 2E). Other KCs marked by CD206 showed (Figure 2F) that few KCs were polarized to M2 KCs. In addition, there was a slight increase in M2 KCs in the IQ group. However, it was unreliable to identify that the anti-inflammatory effect of IQ was achieved by increasing the polarization into M2 KCs. These results suggested that the anti-inflammatory effect of IQ was independent of the classical polarization process of M0 to M2 KCs. Hence, it is required to further investigate the novel mechanism of IQ in achieving the anti-inflammatory effect in another way.

### 2.3. Transcriptomics Study on the Liver Tissue of MCD-Induced Mice

**Figure 3:**
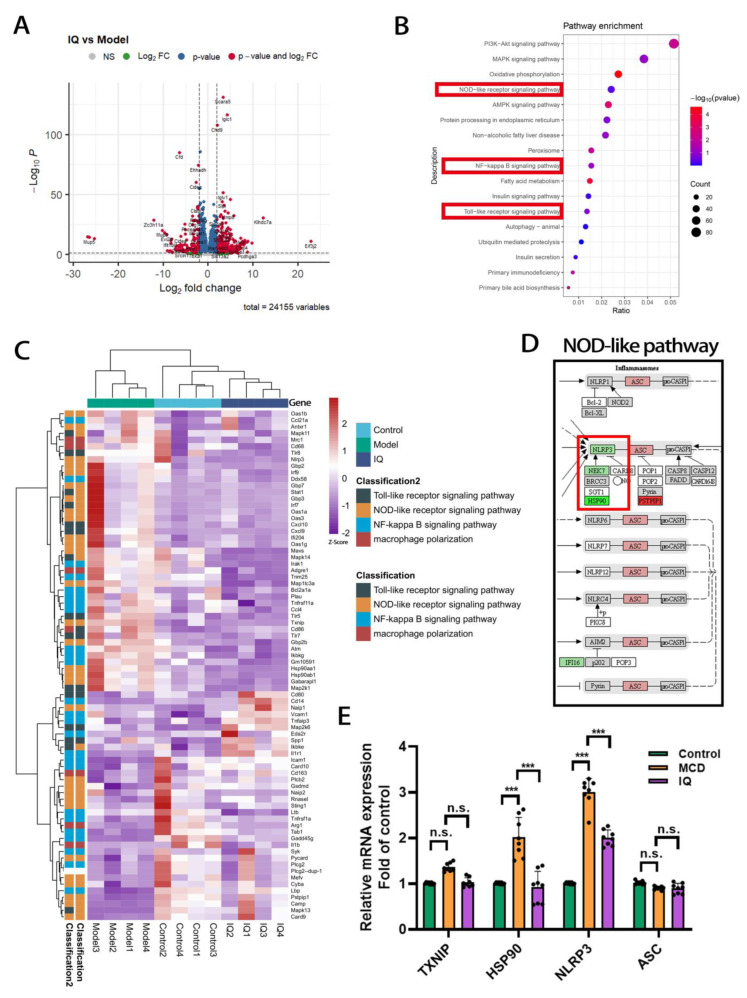
**Transcriptomics study on the liver tissue of MCD-induced mice.** (**A**) Volcano analysis between the IQ and model groups. (**B**) Pathway enrichment of KEGG. (**C**) Heat map of gene expression in 4 inflammation-related pathways. (**D**) Gene expression of the NOD-like pathway. (**E**) Relative mRNA expression of TXNIP, HSP90, NLRP3, and ASC (n = 8, n indicates the number of samples). The bar indicates mean ± SD. n.s. *p >* 0.05, *** *p* < 0.001 represents the significance of differences, and *p* < 0.05 is considered statistically significant. The significance is calculated using ANOVA, followed by Turkey’s test or the Kruskal–Wallis test for the nonparametric test.

Due to a negative result revealed in the KC polarization test, it is necessary to explore a more comprehensive way to delve into the undercover mechanism of the anti-inflammatory effect of IQ. Transcriptomics was conducted to plot the whole picture of the mRNA expression in the liver tissue of mice. A total of 24,155 variables were read out. In addition, differentially expressed genes (DEGs) between the IQ group and the MCD group were analyzed. These DEGs were visualized separately in a volcano plot (Figure 3A). The Kyoto Encyclopedia of Genes and Genomes (KEGG) pathway enrichment was performed to summarize the main functional differences between both groups, which may provide clues for revealing the action mechanism of IQ. From the results (Figure 3B), it was easy to figure out that three major inflammatory-related pathways were highly enriched, including the NOD-like receptor signaling pathway, NF-kB signaling pathway, and TLR signaling pathway. Moreover, a heatmap was employed to cluster the DEGs of these three pathways in the control, MCD, and IQ groups. The cluster results showed that a cluster with higher relevance appeared between the control and IQ groups. This may indicate that the anti-inflammatory effect of IQ was achieved under the mechanism of these three pathways. To identify the factors that played the most significant role in the action mechanism of IQ, Pathview was employed to mark all DEGs in the map (Figure 3D and Figure S1E). Surprisingly, only NLRP3 inflammasome was reduced by IQ in all 8 inflammasomes of the whole map. This result was confirmed by the RT-qPCR test for the related mRNA expression of TXNIP, HSP90, NLRP3, and ASC. This finding provided a specific direction for further research.

### 2.4. Effects of IQ on the HSP90-NlLRP3 Pathway

**Figure 4:**
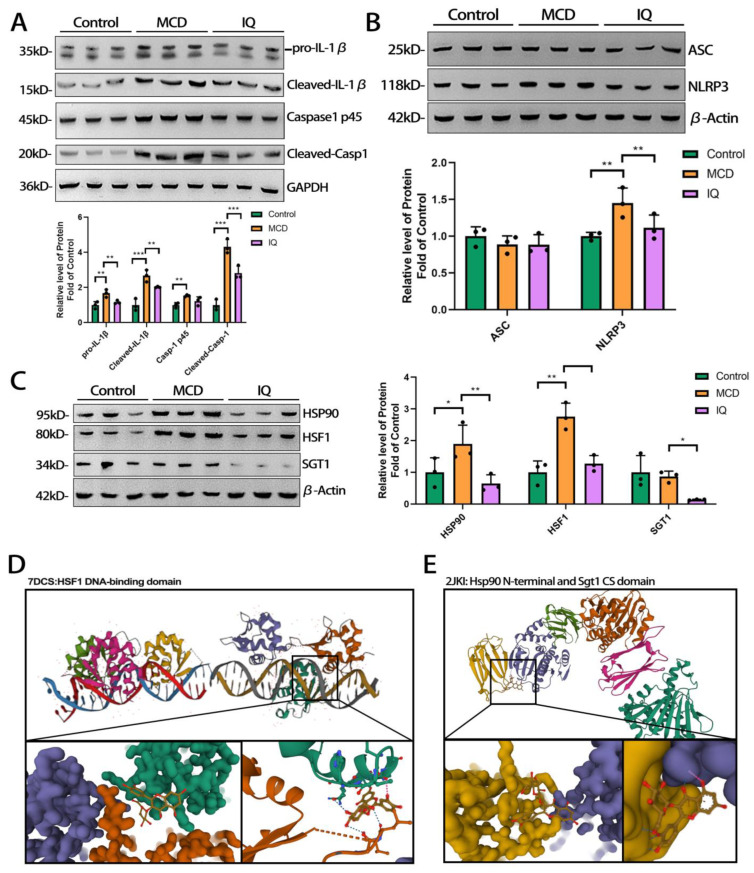
**Effects of IQ on the HSP90-NlLRP3 pathway.** (**A**) Protein expression of pro-IL1β, cleaved-IL1β, caspase-1 and cleaved-caspase-1 in the liver tissue of mice. (**B**) Protein expression of ASC and NLRP3 in the liver tissue of mice. (**C**) Protein expression of HSP90, HSF1, and SGT1 in the liver tissue of mice. (**D**) Molecular docking of IQ with HSF1 and (**E**) HSP90-SGT1. The bar indicates mean ± SD. * *p* < 0.05, ** *p* < 0.01, or *** *p* < 0.001 represents the significance of differences, and *p* < 0.05 is considered statistically significant. The significance is calculated using the Kruskal–Wallis test for the nonparametric test.

Based on the transcriptomics results related to the NLRP3 inflammasome, we first measured the expression of pro-IL-1β and caspase-1, two major proteins secreted by the activated NLRP3 inflammasome, which can result in further inflammatory responses. It was found that caspase-1 and IL-1β secretion were effectively suppressed by IQ (Figure 4A). To further test the activation of NLRP3, the cleaved forms of IL1β and caspase-1 were also tested. The results (Figure 4A) showed that IQ suppressed the expression of both cleaved-IL1β and cleaved-caspase-1, which indicates that the immunoreactivity of NLRP3 was decreased by IQ. Subsequently, the inhibitory effect of IQ on NLRP3 inflammasome activation was investigated. The apoptosis-associated speck-like protein containing a CARD (ASC) is a key element in NLRP3 inflammasome activation. However, the results (Figure 4B) showed that IQ just suppressed the expression of NLRP3, rather than that of ASC. On that basis, it is necessary to explore a novel mechanism of IQ for inhibiting the activation of NLRP3 inflammasomes. From the transcriptomics results (Figure 3D), the gene expression of HSP90 was down-regulated by IQ. HSP90 and SGT1 are the two main proteins of the HSP90–SGT1 protein complex that can regulate the expression of NLRP3. We further measured the protein expression of HSP90 and SGT1. HSF1 is a transcriptional regulation factor in charge of the expression regulation of HSP90. We also examined the protein expression of HSF1. Interestingly, the expression of all these three proteins was suppressed by IQ (Figure 4C). We further utilized docking to simulate the combination of IQ with those proteins. The results showed (Figure 4D) that IQ had a tight link with the HSF1 protein in the area near the DNA-binding domain. This may be the reason that IQ can suppress the expression of HSF1 and HSP90. Furthermore (Figure 4E), the strong association between SGT1 and HSP90 may also suggest that IQ could affect the binding of HSP90–SGT1 complexes and further regulate NLRP3 expression. We then examined the expression of HSP90 in hepatocytes (AML-12) and macrophages (Ana-1) of mice to identify whether IQ can affect different types of liver cells. The results (Figure S1F–H) demonstrated that IQ suppressed the expression of HSP90 in hepatocytes and macrophages.

## 3. Discussion

Recently, some researchers have revealed that the NLRP3 inflammasome plays a crucial role in the progression of NASH. In clinical studies, it can be found that the NLRP3 inflammasome is activated in NASH patients, and it is further associated with the secretion of caspase-1-dependent IL-1β. In animal studies, the expression of NLRP3 is up-regulated in the liver of NAFLD mice, and deficiencies in NLRP3 could inhibit the progression of NASH. These studies further confirm the important role of NLRP3 inflammasome activation in NASH pathogenesis [24,25,26]. Furthermore, it has been demonstrated that the expression of NLRP3 inflammasome complexes is up-regulated in the liver of NASH mice. The treatment with NLRP3 selective inhibitors could mitigate NAFLD and fibrosis in the model of methionine-choline-deficient (MCD) diet-fed mice [27,28]. Moreover, the MCD-fed model provides a histological hallmark of NASH owing to its vulnerability to transition from simple steatosis to steatohepatitis. Therefore, we chose the MCD-induced NASH model to explore the effect of IQ on NLRP3. Interestingly, those phenomena described above were similarly observed in this study, which further confirmed the feasibility of MCD-induced NLRP3 in NASH mice. For now, several active compounds targeting the NLRP3 inflammasome have shown efficacy in treating several diseases in various animal models. However, none of them are used in clinical practice [29,30,31,32,33,34]. Given the potent inflammatory potential of NLRP3 and its role in NASH pathogenesis, targeting NLRP3 inflammasome has been proven to be an attractive pharmacological approach for the pharmacotherapy of NASH. In this study, the transcriptomics study showed that PI3K-Akt, MAPK, and oxidative phosphorylation were enriched at the top of the enrichment result (Figure 3B). This implied that IQ may have an effect on lipid regulation through insulin resistance, autophagy, and anti-oxidative and apoptosis-related mechanisms through PI3K and MAPK in this animal model. This finding was similar to the results of previous studies on quercetin [35,36,37]. These results further confirmed that IQ may exert positive effects at an early stage of NAFLD [23]. However, in this study, we mainly aimed to investigate the anti-inflammatory mechanism of IQ; the inflammation-related NOD-like receptor pathway and NLRP3 were revealed in the results (Figure 3B,D). Hence, it is necessary to conduct further explorations into the NLRP3 inflammasome.

NLRP3 inflammasomes are multimeric protein complexes that are mainly expressed in the liver and assemble in cytosols after sensing DAMPs (such as ROS, inflammatory cytokine, and cholesterol crystals) [38,39]. NLRP3 contains an N-terminal pyrin domain (PYD). This domain is associated with ASC, and it can also recruit pro-caspase-1 to the inflammasome [39,40]. NLRP3 inflammasomes can recruit inactive pro-caspase-1, and the autoproteolytic cleavage of caspase-1 can be induced after its activation by oligomerization pro-caspase-1 proteins. Activated caspase-1 is a cysteine-dependent protease that can cleave the precursor cytokine (pro-IL-1β), thus inducing the secretion of the bioactive cytokine (IL-1β) from cells [41,42,43]. IL-1β further accelerates the inflammation reaction in the liver. The NLRP3 inflammasome is mainly expressed in innate immune cells, including KCs and monocyte-derived macrophages, but rarely in nonimmune cells, such as hepatocytes and hepatic stellate cells [8,25,27,44]. In general, macrophages (KCs) have a lower threshold for inflammasome activation compared with hepatocytes. Hence, the M1 polarization of KCs or the recruitment of macrophages can be first observed at an early stage of liver injury [25]. In this study, we observed that IQ reduced the number of KCs (Figure 1F) in the liver of mice and suppressed the M1 polarization of KCs (Figure 2E). It has been revealed that inhibiting the NLRP3 signaling could attenuate macrophage-related inflammation and inhibit M1 polarization of macrophages in NASH [45]. Moreover, the suppressive effect of IQ on both the total and cleaved form of IL-1β and caspase-1 has indicated that IQ could reduce the activation of NLRP3 inflammasomes (Figure 4A) in the liver of mice, and this may reveal the action mechanism in reducing M1 polarization and KCs.

Normally, free cytosolic NLRP3 is maintained in an inactive ubiquitinated state. Stimulating signaling to deubiquitinate its LRR domain is a well-known process for activating NLRP3 [46,47]. HSP90 is a multifunctional molecular chaperone that can regulate the stabilization and activation of its client proteins involved in immunity responses, signal transduction, protein transport, and receptor maturation [48]. NLRP3 is also the client protein regulated by HSP90. HSP90 is important for maintaining the priming threshold, without which the activation of NLRP3 inflammasomes cannot be achieved. The HSP90–SGT1 complex is a ubiquitin ligase-associated protein. The HSP90–SGT1 complex keeps the NLRP3 in a mature and signal-receptive state. HSP90-SGT1 is thought to be separated from NLRP3 under priming conditions to allow for inflammasome activation [49]. Previous studies have shown that the inhibition of HSP90 can reduce NLRP3 inflammasome activity in macrophages in the liver [50]. Suppressing the activation of NLRP3 inflammasomes in KCs can ameliorate hepatic steatohepatitis [51]. Therefore, inhibiting HSP90-SGT1 could affect the stability of free NLRP3 and interfere with inflammasome activity, which might be a novel way for IQ to have an effect on steatohepatitis. In this study, IQ reduced the expression of both HSP90 and SGT1. Moreover, the docking results revealed that IQ bound tightly in the domain of the HSP90–SGT1 complex (Figure 4E), which could affect the connection between HSP90 and SGT1. The inhibition of HSF1 by IQ may explain the decrease in HSP90, which was caused by reduced transcriptional regulation (Figure 4C). However, a ubiquitination test of NLRP3 or a protein–protein interaction test between HSP90-SGT1 was not performed in this study. IQ is well known as a wide-range target and an anti-oxidative compound inside cells, including reducing ROS and activating Nrf2. Furthermore, it can also inhibit NLRP3 [21]. Moreover, a reasonable and new mechanism was summarized in this study, namely that inhibiting the activated NLRP3 inflammasome through HSP90 by IQ could mitigate steatohepatitis of NAFLD.

## 4. Materials and Methods

### 4.1. Reagents

Isoquercitrin (purity > 99%) was purchased from Chengdu Push Biotechnology Co., Ltd. (Chengdu, China). The silibinin (35 mg/pill) was purchased from Tasly (Tianjin, China).

### 4.2. Treatment of Animals and Transcriptomics Study

Methionine-choline-deficient (MCD) diet (TP 3005G) and normal diet (LAD0020) were purchased from Trophic Co., LTD (Nantong, China). Thirty-two male C57BL/6 mice (18 to 22 g) were purchased from Cavens Laboratory Animal Co., Ltd. (Changzhou, China) and kept in a barrier facility for SPF laboratory animal conditions. After acclimatization for 3 days, mice were randomly divided into 4 groups (n = 8) as follows: control group fed with normal diet and vehicle (CMC-Na+); MCD group fed with MCD diet; IQ group fed MCD and IQ (25 mg/kg/day); and Silibinin group (SIL) administered MCD and silibinin (54.6 mg/kg/day). All the animal study was approved by Ethical Committee of China Pharmaceutical University and Laboratory Animal Management Committee of Jiangsu Province (SYXK(SU)2021-0011, approved 25 January 2021). All the experiments followed the Jiangsu Provincial standard ethical guidelines in using experimental animals under the ethical committees mentioned above. After all the experiments were finished, the hepatic tissues were collected immediately and stored in liquid nitrogen. Two animals’ tissues were put in one tube as a sample for sequencing; therefore, there were 4 samples in each group. Transcriptomics sequencing service was provided by Wekemo Tech Group Co., Ltd. (Shenzhen, China). The data were analyzed on the free online platform Wekemo Bioincloud (Shenzhen, China).

### 4.3. Biochemical Measurement

TC was investigated by commercially available kits (BHKT, Beijing, China). Serum and hepatic quantities of superoxide dismutase (SOD) and malondialdehyde (MDA) were analyzed using commercial kits from Nanjing Jiancheng Bioengineering Institute, Nanjing, China. Quantitation of reactive oxygen species (ROS) was detected using Reactive Oxygen Species Assay Kit (BeyoTime, Shanghai, China), following the manufacturer’s instructions. All the quantitation of above kits was read by a multifunctional microplate reader (BioTek, Winooski, VT, USA).

### 4.4. Histopathological Staining and Immunohistochemical Analysis

Liver tissues were fixed in 10% formalin and processed for hematoxylin–eosin (H&E) staining, Oil Red O staining and Sirius Red staining followed the standard procedure by Microworld Biotech, Co., Ltd. (Nanjing, China). Additionally, CD68 (ab283654, Abcam, Cambridge, UK), F4/80 (ab6640, Abcam), CD206 (ab64693, Abcam), iNOS (ab178945, Abcam), and HSP90 (ab59459, Abcam) anti-bodies were used for immunofluorescent staining. All the staining data were captured by fluorescence microscope (Nikon Ni-U, Shanghai, China).

### 4.5. Real-Time Quantitative PCR (RT-qPCR) Analysis

Hepatic tissues were used for the extraction of total RNA using Trizol reagent (Invitrogen, Waltham, MA, USA). Reverse transcription was performed by HiScript II Q RT SuperMix (Vazyme, Nanjing, China) for the synthesis of cDNA. The qPCR was performed on the StepOnePlus Real-Time PCR System (Applied Biosystems, Waltham, MA, USA) by adding the ChamQTM Universal SYBR qPCR Master Mix (Vazyme, Nanjing, China) and following the manufacturer’s protocol. The specific sequences of primers used in this study were synthesized by Genscript Co., Ltd. (Nanjing, China) and are shown in (Table 1). The 2^−∆∆Ct^ method was used to calculate the expression levels of each of the targeted mRNAs by normalizing to GAPDH.

### 4.6. Western Blotting

The protein suspension of liver tissues for Western blotting was obtained using a total protein extraction kit (APPLYGEN P1250, Beijing, China) and protein concentrations were determined by BCA protein assay kit following the manufacturer’s instructions. CD68 (ab283654, Abcam), HSP90 (ab59459, Abcam), HSF1 (ab242138, Abcam), SGT1 (11019-2, Proteintech), IL-1β (ab234437, Abcam), Caspase 1 (ab179515, Abcam), cleaved-IL-1β (#63124, CST), cleaved-caspase-1 (#89332, CST), NLRP3 (ab263899, Abcam), ASC (#67824, CST), GAPDH (#5174, CST), and β-actin (sc-69879, Santa Cruze) anti-bodies were used and blots were quantified using Image J Software (NIH, Bethesda, MD, USA). Each group was tested using three samples that were created by mixing the liver samples from mice numbered 1–3, 4–6, and 7–8, respectively. Western blot results were normalized to the GAPDH band or the β-actin band.

### 4.7. Statistical Analysis

Statistical analysis was expressed as mean ± SD and performed by Graph Pad PRISM (Graph Pad Software, Boston, MA, USA). Data were analyzed by unpaired two-tailed Student’s *t*-test or by one-way ANOVA with Turkey’s test. The immunohistochemical, PRC, and WB data used Kruskal–Wallis test for the nonparametric test. The differences between groups were considered statistically significant at *p*-value < 0.05.

## 5. Conclusions

In conclusion, IQ can reduce hepatic lipids and exert antioxidant and anti-inflammatory effects in the MCD-induced mice model. In addition, the reduced M1 polarization of KCs was found in the liver of mice. The transcriptomics study showed that the NOD-like pathway was suppressed by IQ and NLRP3 was the key factor suppressed by IQ. Further RT-qPCR and Western blotting results indicated that the NLRP3 inflammasome-related proteins and HSP90-SGT1 were also suppressed by IQ. Hence, it can be concluded that IQ could alleviate steatohepatitis of NAFLD by inhibiting the activated NLRP3 inflammasome by suppressing the expression of HSP90. These findings are expected to provide a novel insight into the IQ mechanism at the steatohepatitis stage of NAFLD.

## Figures and Tables

**Table 1:** Specific sequences of primers used in RT-qPCR.

Name	GenBank Accession	Forward Primer	Reverse Primer
**Keap1**	NM_016679	CGGGGACGCAGTGATGTATG	TGTGTAGCTGAAGGTTCGGTTA
**HO1**	NM_010442	AGGTACACATCCAAGCCGAGA	CATCACCAGCTTAAAGCCTTCT
**TNFa**	NM_013693	CAGGCGGTGCCTATGTCTC	CGATCACCCCGAAGTTCAGTAG
**IL1b**	NM_008361	GAAATGCCACCTTTTGACAGTG	TGGATGCTCTCATCAGGACAG
**IL6**	NM_031168	TAGTCCTTCCTACCCCAATTTCC	TTGGTCCTTAGCCACTCCTTC
**Arg1**	NM_007482	CTCCAAGCCAAAGTCCTTAGAG	AGGAGCTGTCATTAGGGACATC
**TGFβ**	NM_011577	CTCCCGTGGCTTCTAGTGC	GCCTTAGTTTGGACAGGATCTG
**TXNIP**	NM_001009935	TCTTTTGAGGTGGTCTTCAACG	GCTTTGACTCGGGTAACTTCACA
**HSP90**	NM_008302	GTCCGCCGTGTGTTCATCAT	GCACTTCTTGACGATGTTCTTGC
**NLRP3**	NM_145827	ATTACCCGCCCGAGAAAGG	TCGCAGCAAAGATCCACACAG
**ASC**	NM_023258	CTTGTCAGGGGATGAACTCAAAA	GCCATACGACTCCAGATAGTAGC
**GAPDH**	NM_008085	TGGATTTGGACGCATTGGTC	TTTGCACTGGTACGTGTTGAT

## Data Availability

The data used to support the findings of this study are available from the corresponding author or the first author upon request.

## Supplementary Materials

The supporting information can be downloaded at: https://www.mdpi.com/article/10.3390/ijms24108795/s1.
