# Supplementary material for: Isoquercitrin Attenuates Steatohepatitis by Inhibition of the Activated NLRP3 Inflammasome through HSP90
Source: Int J Mol Sci. 2023 May 15;24(10):8795. doi: 10.3390/ijms24108795 (PMC10218527; doi:10.3390/ijms24108795)
Supplement: Supplementary file 1 [file ijms-24-08795-s001.zip › ijms-2337663-supplementary.pdf]

Supplementary Materials

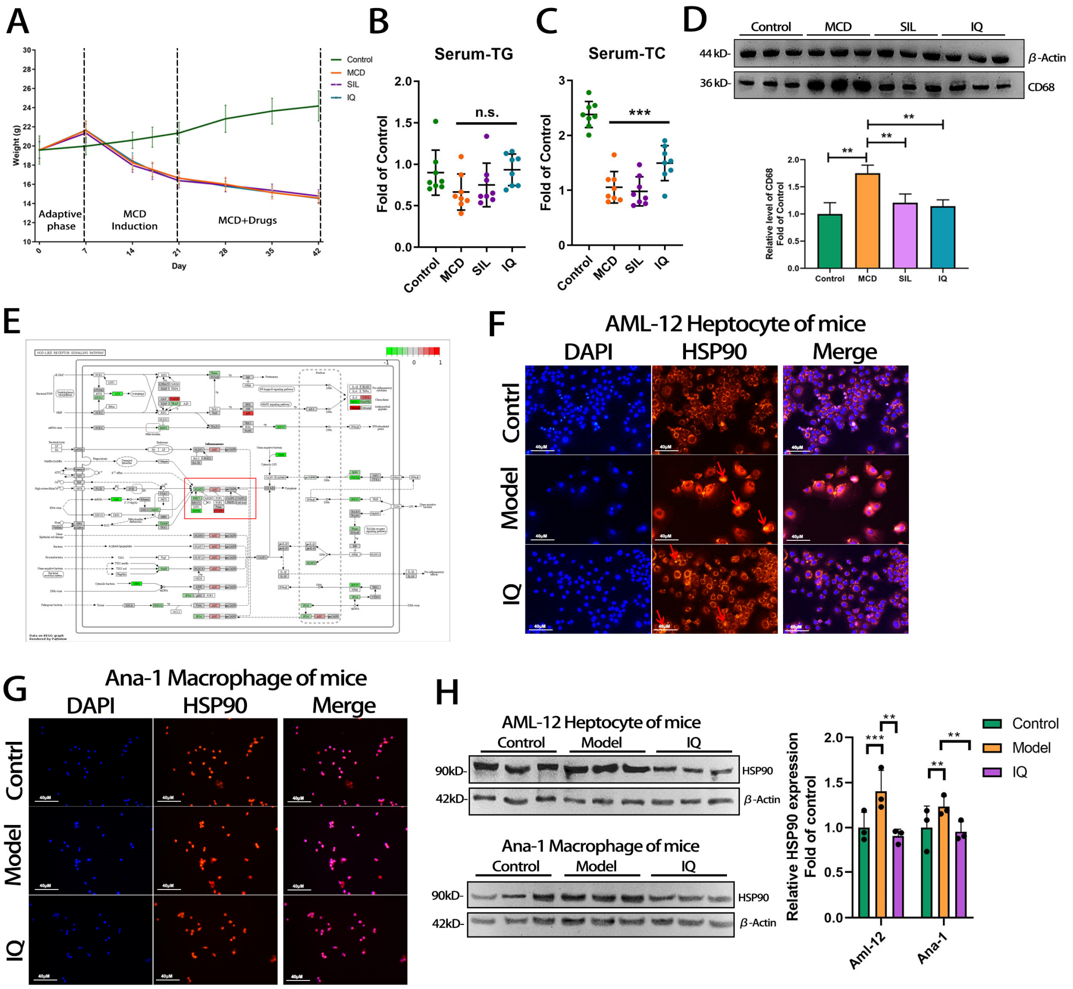

Figure S1. supplementary data. (**A**) Weight of mice. (**B**) Serum TG of mice. (**C**) Serum TC of mice. (**D**) Relative protein expression of CD68 of the liver tissue of mice. (**E**) Gene expression of the NOD-like pathway. (**F**) Immunofluorescence expression of HSP90 in AML-12 cells. (**G**) Immunofluorescence expression of HSP90 in Ana-1 cells. (**H**) Relative protein expression of HSP90 in two cells. The bar indicates mean ± SD. n.s. $p > 0.05$, ** $p < 0.01$, or *** $p < 0.001$ represents the significance of differences, and P<0.05 is considered as statistically significant. The significance is calculated by ANOVA, followed by the Turkey's test or the Kruskal-Wallis test for the nonparamteric test.
